# Left Cerebellar Stroke in a Patient With Parkinson's Disease Complicated With Prolonged Position-Related Compression Syndrome: A Case Report

**DOI:** 10.7759/cureus.88798

**Published:** 2025-07-26

**Authors:** Anastasiia K Shkvarok-Lisovenko, Yaroslava Korost, Liubov P Sokolova, Olha A Ilina, Yehor Lisovenko

**Affiliations:** 1 Department of Family Medicine, Bogomolets National Medical University, Kyiv, UKR; 2 Department of Anesthesiology and Reanimation, Kyiv City Clinical Hospital №8, Kyiv, UKR; 3 Department of Neurology, Bogomolets National Medical University, Kyiv, UKR

**Keywords:** necrosis, parkinson's disease, pressure sores, prolonged compression syndrome, stroke

## Abstract

Lonely living among the elderly has become a widespread issue in Ukraine, particularly in the context of the mass emigration of the younger generation seeking safety and stability abroad. This demographic shift leaves many older adults vulnerable and often incapable of ensuring adequate self-care or timely medical assistance in emergency situations.

Individuals with Parkinson's disease may be at increased risk for stroke. In cases of solitary living, the prognosis for such patients remains particularly poor.

This case report presents an elderly patient with Parkinson's disease who experienced an acute cerebrovascular accident complicated by prolonged compression positional syndrome, resulting from delayed medical intervention due to solitary living.

## Introduction

Parkinson's disease (PD) is a complex, progressive neurodegenerative disorder of the nervous system that primarily affects movement. It is characterized by tremor, muscular rigidity, and bradykinesia, with postural instability developing in some patients as the disease advances. The condition results from the gradual weakening, damage, and loss of neurons in specific areas of the brain, particularly the substantia nigra, leading to hallmark motor symptoms, such as impaired balance, stiffness, and resting tremors [1].

Postural instability and gait disturbances, which are core symptoms of PD, significantly increase the risk of falls, affecting between 35% and 90% of patients and posing a major challenge in disease management [2].

Recent studies show a growing prevalence of PD in the last decades in Ukraine [3]. Stroke care in Ukraine remains a critical public health concern, with approximately 130,000 cases reported annually and mortality rates exceeding the World Health Organization (WHO) European average. After reaching its lowest incidence in 2022 (82.1 per 100,000), stroke cases rose by 22.4% in 2023 (to 100.5 per 100,000), while hospitalizations increased by 26.6% compared to 2016, largely due to recurrent strokes. Furthermore, the ischemic-to-hemorrhagic stroke ratio shifted markedly, from 8.4:1 in prewar years to 12.7:1 during the period of martial law, reflecting changes in patient profiles and healthcare access amidst ongoing conflict [4]. Due to the elevated fall risk among individuals with PD, independent living without immediate access to support substantially increases their vulnerability to injury, including the development of pressure sores and crush syndrome as a result of prolonged immobility in a single position. Chronic exertional compartment syndrome refers to the traumatic rhabdomyolysis leading to a spectrum of disorders culminating in acute kidney injury [5].

This case report aims to highlight the severe consequences and atypical complications of ischemic stroke in elderly individuals living alone, which is particularly widespread in the context of large-scale youth emigration from the country [6-10].

## Case presentation

**Figure 1:**
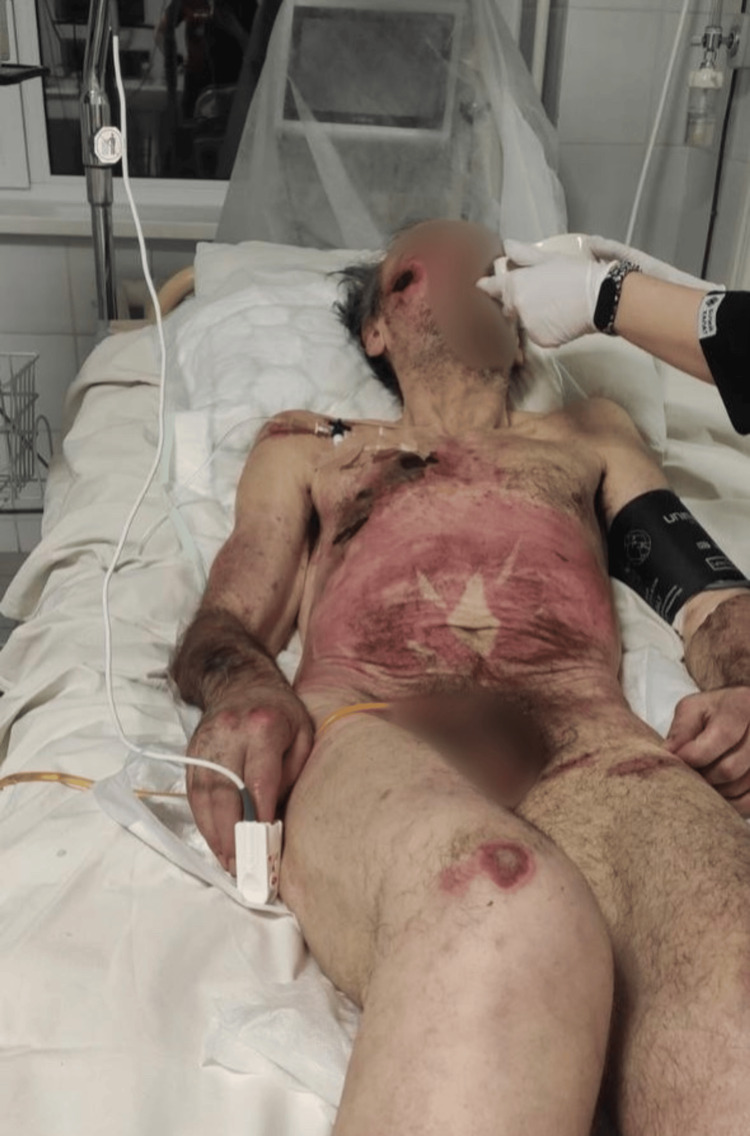
Extensive infected wounds on the patient's skin resembling decubitus ulcers with areas of necrosis and maceration involving the facial area, trunk, and all extremities

A 62-year-old male patient was referred to the Kyiv City Clinical Hospital Emergency Department for further management in the Department of Purulent Surgery, having initially presented to the emergency department of another healthcare facility. Upon admission, the working diagnosis included positional compression syndrome; multiple infected wounds resembling pressure ulcers with areas of necrosis and maceration involving the facial area, trunk, and all four extremities; acute kidney injury; and electrolyte imbalance syndrome (Figure 1). Given the patient's critical condition, he was admitted directly to the intensive care unit (ICU) for further stabilization and management.

His medical history was notable for PD for approximately seven years and grade III essential hypertension, stage 3, risk category 4, diagnosed 10 years earlier. The patient experienced an inferior wall and interventricular septal myocardial infarction in 2022. He had been on regular pharmacologic therapy, including biperiden 1 mg twice daily. No additional pharmacologic agents were reported in the patient's medication history.

According to the patient's history, the acute event began with the sudden onset of profound generalized weakness, resulting in a forward fall in the corridor of his apartment, where he lives alone. All of the patient's relatives emigrated for permanent residence abroad following the onset of the military invasion of Ukraine in 2022. Unable to rise due to neuromuscular dysfunction, he remained conscious but immobilized on the floor for approximately 72 hours. He was eventually discovered by emergency medical services, alerted by neighbors who noticed an unusual and foul odor emanating from the apartment.

**Figure 2:**
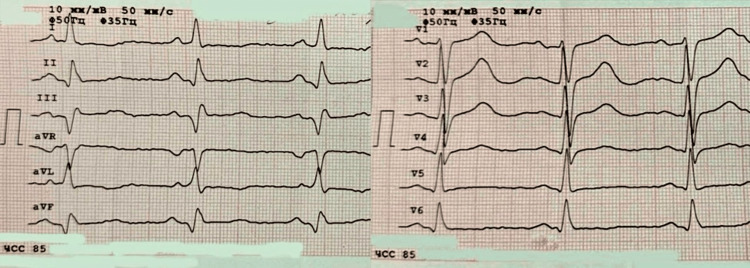
Primary ECG of the patient The electrocardiogram was recorded at a paper speed of 50 mm/s and a standard amplitude of 10 mm/mV. The rhythm is sinus: upright P waves are visible in all leads (I, II, III, aVR, aVL, aVF, V1-V6), each preceding a QRS complex, with a regular rhythm and a normal PR interval. The heart rate is 85 beats per minute. Pathological Q waves are present in the inferior leads II, III, and aVF, indicating a previous myocardial infarction of the inferior (diaphragmatic) wall. The ST segments are isoelectric across all leads, with no elevation or depression, excluding evidence of acute ischemia at the time of recording. ECG: electrocardiography

Upon initial assessment in the first medical facility, his condition was classified as severe but hemodynamically stable. Vital signs revealed hypotension (blood pressure (BP) 60/40 mmHg) and tachycardia (heart rate (HR) 85-120 bpm). Given elevated troponin I levels (3.38 ng/mL; reference <0.03 ng/mL), acute coronary syndrome (ACS) was suspected. An electrocardiography (ECG) was performed, which showed no typical signs of ACS (Figure 2).

**Figure 3:**
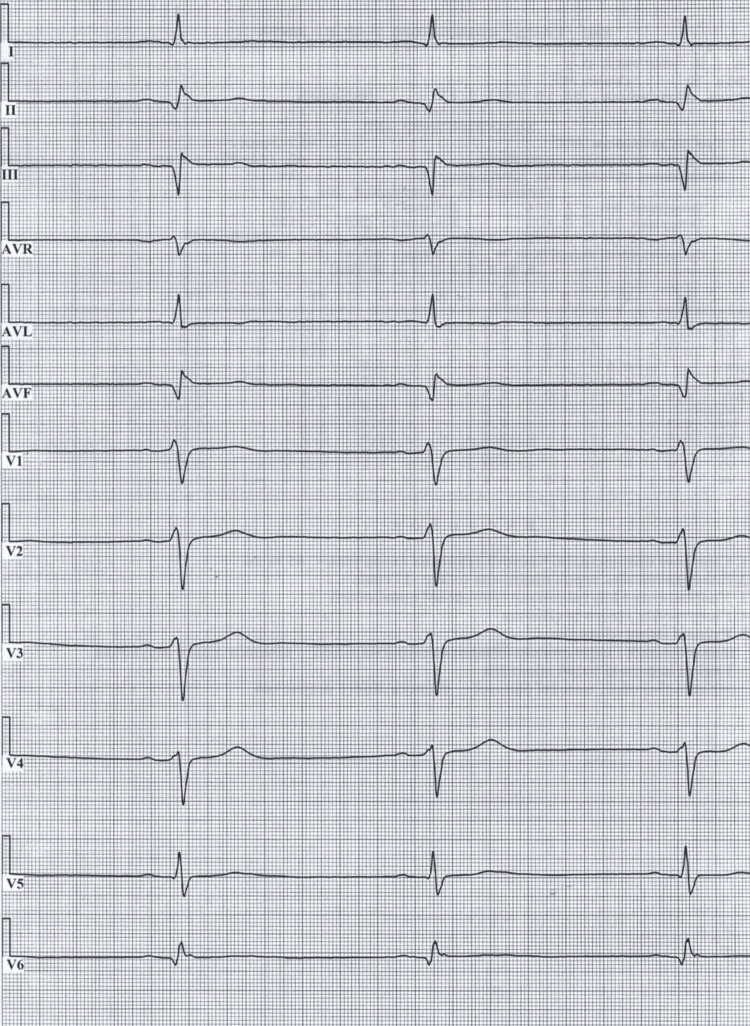
Repeat ECG of the patient The electrocardiogram was recorded at a paper speed of 50 mm/s and a standard amplitude of 10 mm/mV. The rhythm is sinus: upright P waves are visible in the all leads, each preceding a QRS complex, with a regular rhythm and normal PR interval. The heart rate is 46 beats per minute. Pathological Q waves are present in the inferior leads II, III, and aVF, indicating a previous myocardial infarction of the inferior (diaphragmatic) wall; the presence of Q waves in the precordial leads V2-V4 accompanied by reduced R-wave amplitude confirms the previous involvement of the interventricular septum. T-wave inversion is observed in leads V2-V4, indicating post-ischemic changes. The ST segments are isoelectric across all leads, with no elevation or depression, excluding evidence of acute ischemia at the time of recording. ECG: electrocardiography

On arrival at the receiving hospital, physical examination revealed extensive soft tissue damage, including multiple infected pressure-type ulcers with necrosis and maceration on the anterior body surface, particularly involving the face, chest, and all extremities. The patient complained of persistent generalized weakness and localized pain in the areas of soft tissue injury. Biochemical studies showed elevated serum creatinine at 141 µmol/L, with an estimated glomerular filtration rate (eGFR) of 44.5 mL/min/1.73 m². He denied chest pain or other cardiovascular complaints. Repeat ECG taken three hours after the first one showed no acute ischemic changes (Figure 3). HR decreased to 46 beats per minute. Any other dynamic changes were not observed in the remaining ECG parameters. Performing echocardiography was necessary, but remained impossible due to a blackout in Kyiv caused by a massive missile attack.

The patient's laboratory results showed leukocytosis (white blood cell (WBC) 14.4 K/µL) with marked neutrophilia (95%) and lymphopenia (3%), suggesting an acute inflammatory response, likely related to tissue damage from prolonged compression or possible secondary infection (Table 1).

**Table 1:** Complete blood count of the patient WBC: white blood cells; RBC: red blood cells; Neu: neutrophils; LY: lymphocytes; Mon: monocytes; Eo: eosinophils; Bas: basophils; ESR: erythrocyte sedimentation rate

Diagnostic caption	Result	Reference range
WBC (K/µL)	14.4	4-11
RBC (T/L)	5.2	3.9-5.2
Hemoglobin (g/L)	150	120-156
Hematocrit (%)	45	35.5-45.5
Platelet count (K/µL)	200	166-389
Neu (%)	95	40-70
LY (%)	3	20-44
Mon (%)	2	2-9.5
Eo (%)	0	0.5-5.5
Bas (%)	0	0-1.75
ESR (mm/hour)	12	<15

Biochemical blood analysis revealed significant abnormalities consistent with rhabdomyolysis and secondary organ involvement in the patient. Muscle injury markers were markedly elevated, namely, creatine phosphokinase (10.300 U/L), lactate dehydrogenase (1,630 U/L), aspartate aminotransferase (299 U/L), and alanine aminotransferase (256 U/L), confirming extensive muscle breakdown. Urea (26.6 mmol/L) and creatinine (141 µmol/L) were significantly elevated, indicating acute kidney injury likely due to myoglobin-induced tubular damage. Electrolytes showed mild hypernatremia (Na⁺ 145.7 mmol/L) and hyperchloremia (Cl⁻ 109.2 mmol/L), suggesting possible dehydration or acid-base imbalance. Liver function tests revealed elevated total bilirubin (27 µmol/L), direct bilirubin (7 µmol/L), and indirect bilirubin (20 µmol/L), along with decreased albumin (27 g/L), pointing to hepatic stress or systemic inflammation. Procalcitonin (0.59 ng/mL) was mildly elevated, possibly reflecting low-grade infection or inflammatory response. Glucose and potassium levels were within normal limits (Table 2).

**Table 2:** Biochemical blood analysis of the patient K⁺: potassium; Na⁺: sodium; Cl⁻: chloride; ALT: alanine aminotransferase; AST: aspartate aminotransferase; LDH: lactate dehydrogenase; CPK: creatine phosphokinase

Diagnostic caption	Result	Reference range
K⁺ (mmol/L)	4.22	3.5-5.1
Na⁺ (mmol/L)	145.7	135-145
Cl⁻ (mmol/L)	109.2	98-107
ALT (U/L)	256	<39
AST (U/L)	299	<37
LDH (U/L)	1630	140-280
CPK (U/L)	10300	38-174
Direct bilirubin (µmol/L)	7	<5
Indirect bilirubin (µmol/L)	20	<13
Total bilirubin (µmol/L)	27	5-21
Urea (mmol/L)	26.6	2.5-8.3
Creatinine (µmol/L)	141	44-97
Total protein (g/L)	65	64-83
Albumin (g/L)	27	35-50
Procalcitonin (ng/mL)	0.59	<0.5
Blood glucose (mmol/L)	5.3	3.9-5.8

Overall, the findings highlighted rhabdomyolysis with renal and hepatic implications, likely triggered by prolonged immobility and compression in a neurologically compromised patient.

A slight decrease in the prothrombin index was observed, whereas the other coagulation parameters were markedly elevated (Table 3).

**Table 3:** Coagulation panel and troponin I level PTI: prothrombin index; INR: international normalized ratio; aPTT: activated partial thromboplastin time

Diagnostic caption	Result	Reference range
PTI (%)	76	80-120
INR	5.3	0.8-1.2
aPTT (s)	147	25-35
Fibrinogen (g/L)	6.2	1.8-4.0
Troponin I (ng/mL)	3.38	<0.04

Urinalysis revealed the presence of leukocyturia, abundant mucus, and a moderate quantity of bacteria (Table 4).

**Table 4:** General urine examination WBC: white blood cells; RBC: red blood cells

Diagnostic caption	Result	Reference range
Specific gravity	1.019	1.010-1.025
Protein (g/L)	0.33	<0.033
Glucose (mmol/L)	0	0
pH reaction	4.5	4.5-8.0
Ketones (semiquantitative)	Negative	Negative
WBC (per high-power field)	6-8	0-5
RBC (per high-power field)	1-3	0-3
Granular casts (per low-power field)	1-2	0-2
Hyaline casts (per low-power field)	0-1	0-2
Mucus (qualitative)	+++	None to trace
Bacteria (per high-power field)	+	Absent

The patient was admitted to the ICU, where the following therapeutic measures were initiated: intravenous (IV) infusion of crystalloids (1500 mL); digoxin 0.025%, 1 mL twice daily IV; ceftriaxone 1 g twice daily IV; omeprazole 40 mg once daily orally; enoxaparin sodium 0.4 mL subcutaneously once daily; and oral enterosorbent, one sachet four times daily.

A cardiology consultation was obtained. According to the absence of typical findings, ACS was ruled out. After exclusion of ACS, the patient was referred for a computed tomography (CT) scan of the brain. A multidisciplinary consultation involving a traumatologist, neurologist, surgeon, and anesthesiologist was conducted to comprehensively evaluate the patient's clinical status.

**Figure 4:**
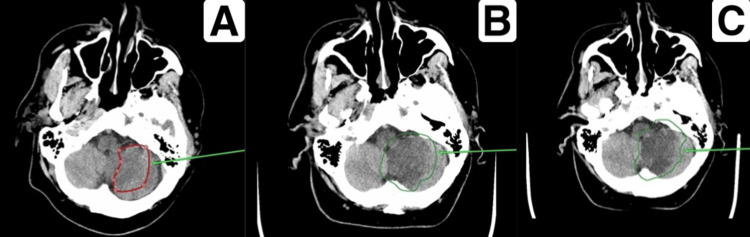
Comparison of the stroke-affected area over time (A) Stroke-affected area as of 22.02.2023. (B) Extension of the affected area as of 23.02.2023. (C) Affected area as of 01.03.2023. The green arrow highlights the region of stroke propagation observed within three days following the patient's admission to the hospital.

The scanning was performed in axial projection, followed by multiplanar reconstruction. In the left hemisphere of the cerebellum, a noted loss of differentiation between white and gray matter was found, with the sulci appearing effaced and gyri swollen. The subarachnoid space in the convexital regions at the level of the lesion was not visible. There was an observed increase in the density of the posterior inferior cerebellar artery (PICA), indicative of thrombosis. The maximum estimated dimensions of the affected area were 43×52×42 mm. On 23.02.2023, the dimensions were 43×50×36 mm, and on 22.02.2023, they were 35×42×36 mm. There is also an area of reduced density at the level of the left cerebellar peduncle in the territory of the anterior inferior cerebellar artery (AICA) and the appearance of areas of reduced density at the level of the left pons, measuring up to 9×7 mm in the territory of the basilar artery (Figure 4).

The subarachnoid space on the convexity surface of the cerebral hemispheres and cerebellum was unevenly expanded. The basal cisterns of the brain were expanded, but not deformed. The lateral fissure on the right was not expanded; on the left, it was accentuated. The gyri of the cerebral hemispheres and cerebellum were moderately atrophic throughout the rest of their extent.

At the level of the basal ganglia, in the white matter of the cerebral hemispheres, there were multiple areas of reduced density with indistinct margins, ranging from 3 mm to 14 mm in diameter, showing no change over time. Diffuse enlargement of the perivascular spaces was noted, up to 5 mm in diameter, with no change over time.

**Figure 5:**
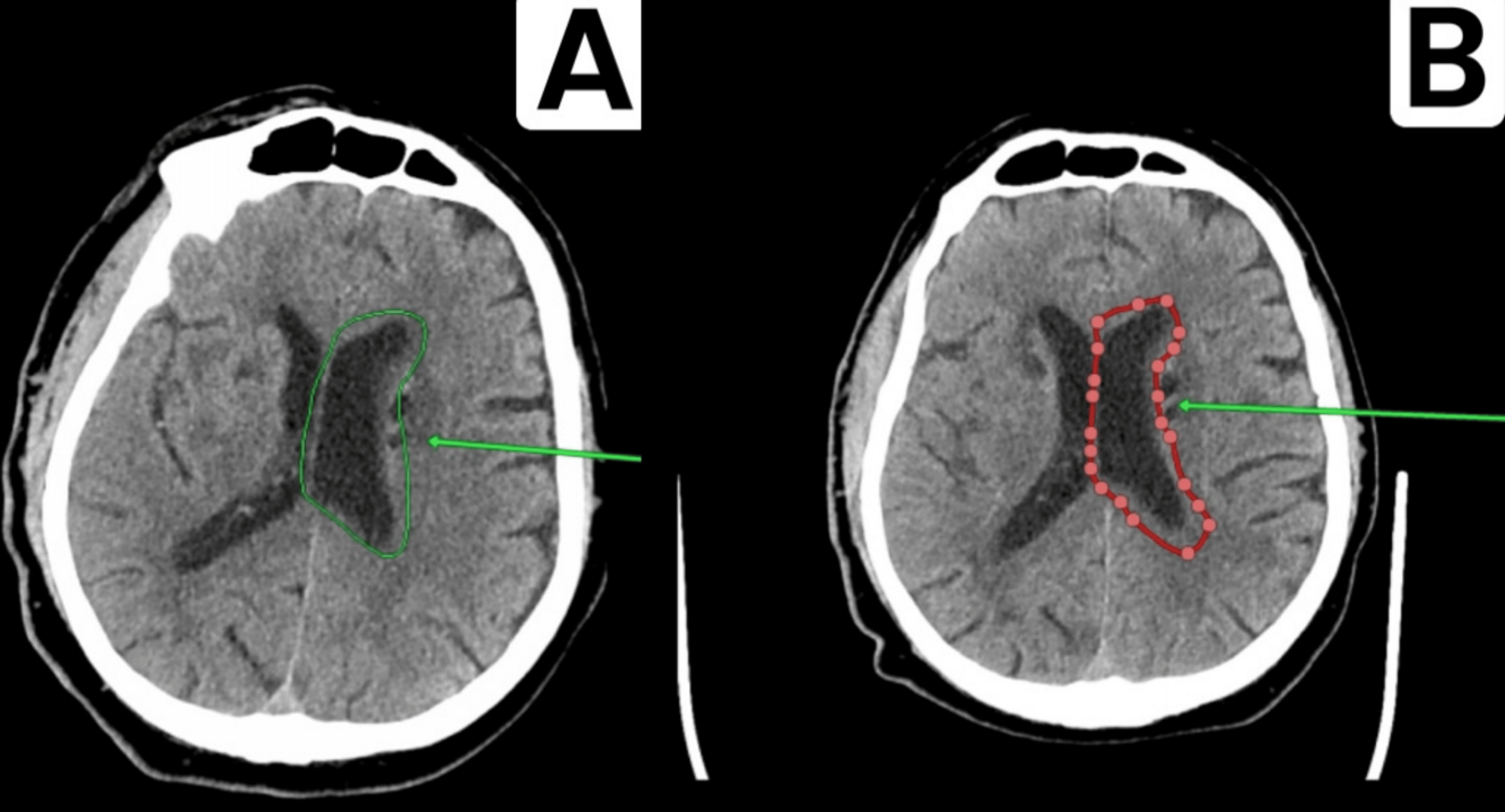
Asymmetry of the brain ventricles. Comparison of left ventricular dimensions over time (A) Left ventricular dimensions as of 22.02.2023. (B) Left ventricular dimensions as of 01.03.2023.

The brain ventricles are asymmetrical but not deformed. The anterior horns at the level of the interventricular (Monro) foramina were 6 mm on the right, with no change, and 12 mm on the left (previously up to 9 mm). The third ventricle measures 12 mm (previously up to 9 mm) (Figure 5).

**Figure 6:**
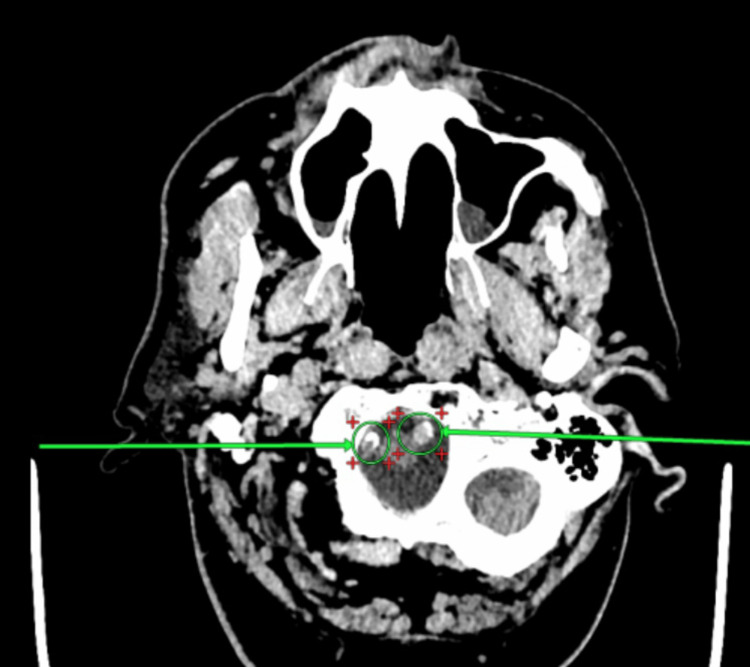
Sclerotic walls of the vertebral arteries

Periventricular white matter demonstrated reduced density (leukoaraiosis), up to 10 mm in width. The midline structures were not displaced. The brainstem, cerebellum, and cerebellopontine angles were unchanged throughout the remainder of their extent. The walls of the internal carotid, vertebral, and basilar arteries were sclerotic, with calcified atherosclerotic plaques (Figure 6). The internal auditory canals were not enlarged and symmetrical, with diameters equal on both sides. No destructive changes were observed in the bones of the skull base and vault. The contents of the orbits were unremarkable, and the retrobulbar space was unchanged. Pneumatization of the visible paranasal sinuses was not impaired.

**Figure 7:**
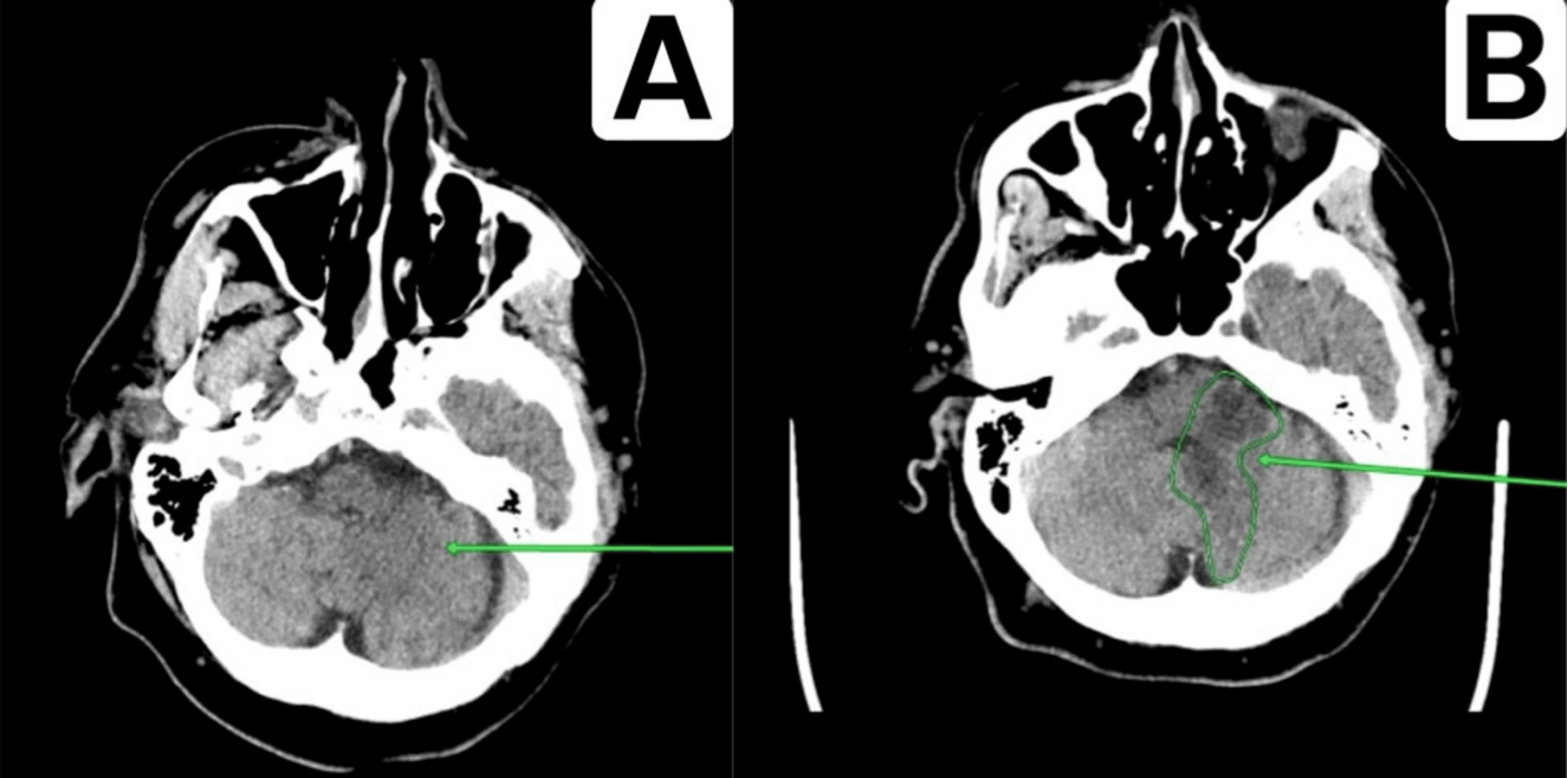
Signs of progression of acute cerebrovascular accident due to the involvement of the territories of the anterior inferior cerebellar artery and the basilar artery on the left (A) Affected area as of 22.02.2023. (B) Affected area as of 01.03.2023.

**Figure 8:**
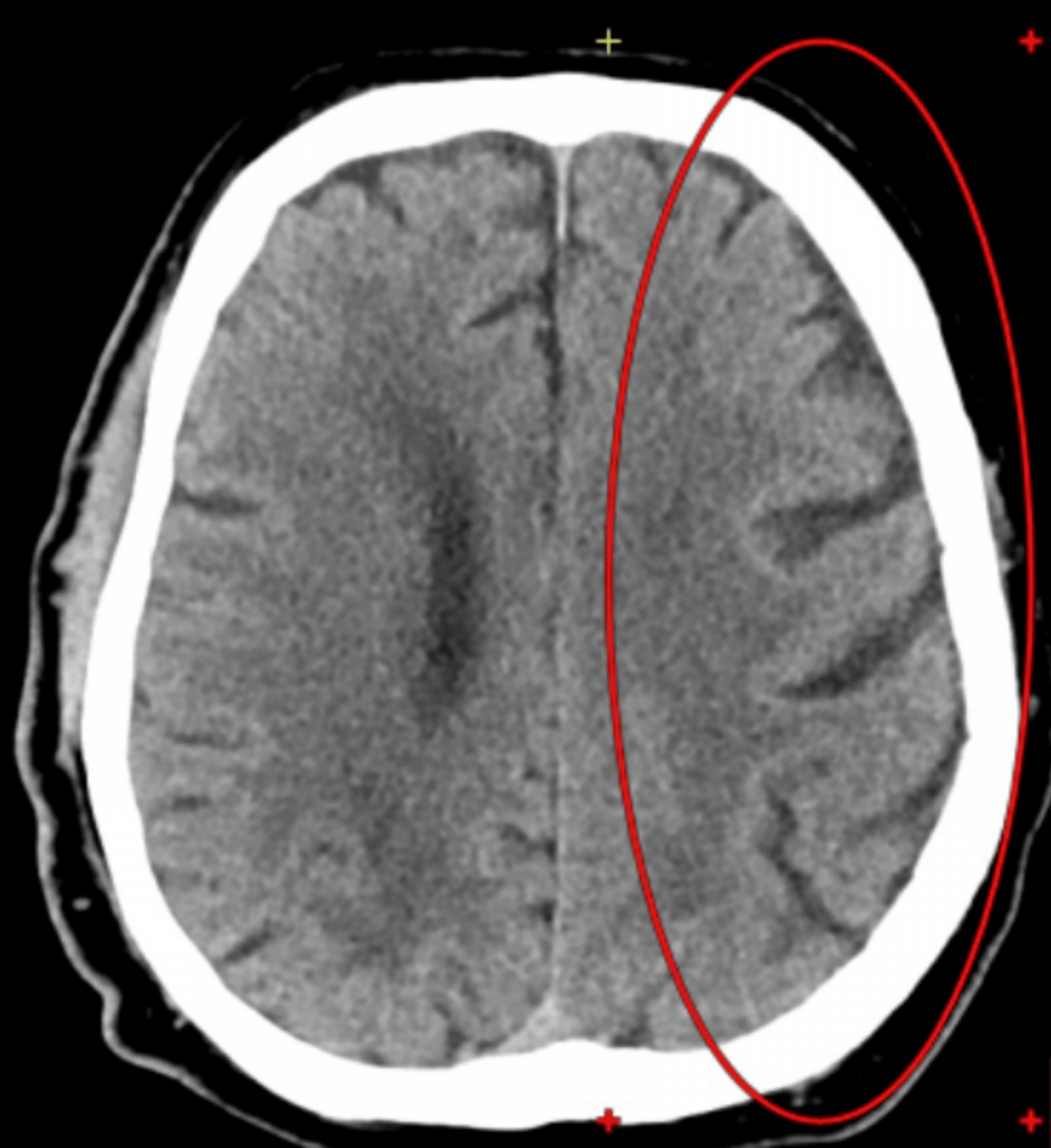
Atrophy of the cerebral and cerebellar hemispheres (GCA grade II)

CT showed signs of stroke of ischemic type in the territory of the PICA, consistent with recent infarction. Compared to the scans from 22.02.2023 and 23.02.2023, there is a progression of CVA phenomena due to the involvement of the territories of the AICA and the basilar artery on the left (Figure 7). CT demonstrated signs of cerebral microangiopathy with focal changes of varying degrees of chronicity, along with generalized atrophy of both cerebral and cerebellar hemispheres, consistent with GCA grade II (Figure 8). 

The patient was transferred to the Department of Neurology, where targeted therapy was initiated, including anticoagulation with enoxaparin sodium 0.4 mL once daily subcutaneously; antithrombotic therapy with clopidogrel 75 mg once daily; intravenous neuroprotective therapy with 25% magnesium sulfate in saline; and maintenance of fluid and electrolyte balance. Surgical debridement of necrotic tissues was planned following neurological stabilization.

The final diagnoses were as follows: acute ischemic stroke in the territory of the left PICA with progression to involve the territories of the AICA and the left basilar artery; cerebral microangiopathy with focal chronic ischemic changes; cerebral and cerebellar atrophy (GCA grade II); positional crush syndrome; multiple infected pressure-type ulcers with necrosis and maceration of the face, trunk, and extremities; acute kidney injury; and electrolyte imbalance syndrome.

The patient was hospitalized in the Department of Neurology for a period of five days. After that, he was transferred to the Department of Purulent Surgery for surgical wound management. He remained under inpatient medical supervision for an additional seven days and was subsequently discharged in satisfactory condition. Upon discharge, the patient was unable to ambulate independently and required the use of a specialized wheelchair.

Following discharge, the patient continued to be monitored by his family doctor. He was prescribed antihypertensive therapy, and the healing of traumatic skin lesions was closely observed.

Within the three months following discharge, the patient visited his family doctor twice, each time accompanied by neighbors. The first visit was related to obtaining a referral for rehabilitation after the cerebrovascular accident, and the second occurred upon the completion of the rehabilitation course, with the purpose of obtaining a referral for evaluation by the Medical and Social Expert Commission to determine his disability group. A significant decline in mobility and a complete loss of self-care ability were documented, indicating the necessity of continuous external assistance. The patient was officially granted disability status.

In the three months following his last visit, the patient did not seek further medical consultation from his family doctor. No medical consultations were recorded for the patient during the two-year period after the incident at all.

## Discussion

This case highlights the multifactorial vulnerability of elderly individuals with PD living alone, particularly during the martial law in Ukraine. The patient suffered an ischemic stroke in the territory of the left PICA, with progression to the AICA and basilar artery regions, as confirmed by CT. Prolonged immobility after a fall led to crush syndrome, pressure ulcers, and acute kidney injury, illustrating the complex interplay between neurodegeneration, vascular pathology, and social isolation.

Stroke remains a major public health issue in Ukraine, with approximately 130,000 cases annually and an increasing incidence since 2022 [4]. Post-stroke complications are exacerbated by a strained healthcare infrastructure and limited access to timely care. PD inherently increases cerebrovascular risk through factors such as hypertension, autonomic dysfunction, and immobility. Falls, due to postural instability, bradykinesia, and freezing of gait, affect up to 80% of patients [11]. In this case, the fall triggered a cascade of complications, including crush syndrome and systemic injury.

Crush syndrome developed as a direct result of prolonged immobility, with rhabdomyolysis leading to acute kidney injury, as evidenced by markedly elevated creatine phosphokinase, aspartate aminotransferase, and lactate dehydrogenase. Metabolic disturbances and ulcer infection further complicated the course.

Beyond medical issues, this case reflects the critical role of social isolation. Delayed discovery and intervention allowed severe complications to develop. With mass emigration, infrastructure damage, and healthcare overload, elderly patients living alone face disproportionate risks [6,7,9].

## Conclusions

This case highlights the complex interplay between neurodegeneration, vascular pathology, pharmacological burden, and social vulnerability in elderly patients living alone. In Ukraine, these factors create a syndemic that heightens the risk of poor outcomes. Timely fall detection, medication review, and the development of community-based support systems are urgently needed to improve care and reduce complications in this high-risk population.
